# Scientific and Public Health Challenges in Folic Acid Supplementation: Insights from Brazil and Global Implications

**DOI:** 10.3390/nu17172752

**Published:** 2025-08-26

**Authors:** Marília Körbes Rockenbach, Ricardo Rohweder, Lavinia Schuler-Faccini, Maria Teresa Vieira Sanseverino, Thayne Woycinck Kowalski

**Affiliations:** 1 Graduate Program in Genetics and Molecular Biology (PPGBM), Genetics Department, Universidade Federal do Rio Grande do Sul, Porto Alegre 91501-970, Brazil; ricardo.rohweder@ufrgs.br (R.R.); lschuler@hcpa.edu.br (L.S.-F.); msanseverino@hcpa.edu.br (M.T.V.S.); 2 Teratogen Information System (SIAT), Medical Genetics Service (SGM), Hospital de Clínicas de Porto Alegre, Porto Alegre 90035-903, Brazil; 3 Laboratory of Genomic Medicine (LMG), Center of Experimental Research, Hospital de Clínicas de Porto Alegre, Porto Alegre 90035-903, Brazil; 4 Medical School, Pontifícia Universidade Católica do Rio Grande do Sul (PUCRS), Porto Alegre 90619-900, Brazil

**Keywords:** folate, 5-methyltetrahydrofolate, dose, pregnancy, health inequalities

## Abstract

Folic acid supplementation during the periconceptional period is a well-established strategy to prevent neural tube defects (NTDs). However, emerging uncertainties surrounding optimal dosage, alternative folate forms, duration of use, and the role of genetic variability have sparked scientific and policy debates. In Brazil, these challenges are amplified by unique contextual factors, including the routine distribution of high-dose folic acid (5000 µg) through the public health system, social vulnerability, low adherence, and limited population-specific evidence for the country’s highly admixed population. This narrative review critically examined the scientific evidence and public health policies surrounding folic acid supplementation, with a focus on Brazil. We examined historical developments, international recommendations, and emerging controversies, including the emerging influence of misinformation. Despite the worldwide progress in NTD prevention, both local and global challenges persist, including delayed initiation, inconsistent adherence, and uncertainty regarding alternative supplementation regimens. Addressing these demands requires the development of context-specific guidelines, alongside coordinated strategies in provider training, public education, monitoring, and research investments to ensure effective, safe, and equitable folic acid supplementation.

## 1. Introduction

Maternal nutrition plays a crucial role in ensuring a healthy pregnancy, supporting both periconceptional health and optimal fetal development, and promoting long-term health outcomes for both mother and child [1]. Among nutritional factors, folate deficiency during the periconceptional period is strongly associated with an increased risk of congenital anomalies, especially neural tube defects (NTDs) [2,3]. The periconceptional period refers to the time surrounding conception; although its exact duration may vary according to the study and context, it is usually defined as the 14 weeks before and 10 weeks after conception, encompassing gametogenesis, fertilization, and early embryonic and placental development [4]. NTDs result from the failure of neural tube closure during early embryogenesis (between 17 and 28 days post-conception), leading to significant morbidity and mortality, depending on the location and severity of the lesion [5]. The specific type of NTD reflects the site of disrupted neurulation. This could range from the most severe form, craniorachischisis, involving the failure of neural tube closure at the initial site, resulting in an open brain and spinal cord, to less severe forms such as spina bifida, caused by incomplete closure of the caudal neural tube [5].

Most NTDs have a multifactorial etiology [6], with maternal folate status representing the principal modifiable environmental determinant risk [7]. Folate, a water-soluble B9 vitamin, is naturally present in foods such as green leafy vegetables and meats and plays a critical role in DNA and RNA synthesis, as well as in DNA methylation, which are fundamental to embryonic development [8]. During pregnancy, the demand for folate increases significantly to support the intense proliferation and differentiation of fetal cells [9]. Given its critical role, folic acid, the synthetic form of folate, has been recommended globally for women of reproductive age. Many countries are adopting mandatory and/or voluntary folic acid fortification policies [10,11], which has significantly contributed to reductions in NTD prevalence worldwide [10,12].

The United States and Canada were the first countries to mandate folic acid fortification in 1996 and 1998, respectively, followed by several Central and South American countries in the late 1990s and 2000s, and by Australia in 2009. The European Union and China introduced voluntary fortification in 2006 and 2012, respectively. A systematic review reported that mean plasma folate concentrations increased from 18.2 to 33.9 nmol/L in the Americas and Australia after mandatory fortification, from 15.2 to 21.4 nmol/L in Europe following voluntary fortification, and from 13.2 to 20.1 nmol/L in China [10]. The same review also showed that the mean prevalence of NTDs per 10,000 births was 4.19 in populations with mandatory fortification, 7.61 in those with voluntary fortification, and 9.66 in populations without fortification [10], underscoring the public health impact of fortification policies.

However, the widespread use of both supplementation and fortification, often without coordinated public health monitoring, has led to the emergence of new concerns. These include potential adverse effects of excessive folate intake, such as possible masking of vitamin B12 deficiency, increased susceptibility to gestational diabetes mellitus, and associations with certain types of cancer [13]. Although these concerns warrant attention, the evidence remains limited and inconclusive. Scientific debates have intensified regarding the optimal dosage and duration of supplementation, particularly beyond the first trimester [13,14,15], as well as the potential benefits of alternative folate forms, such as 5-methyltetrahydrofolate (5-MTHF), especially in individuals with genetic polymorphisms that impair folic acid metabolism [16,17]. While these discussions are valuable for advancing knowledge, their communication to the public is sometimes concerning. Sensationalist headlines and oversimplified interpretations fuel misinformation. Claims that high folate levels cause autism spectrum disorder or that 5-MTHF is superior to folic acid, despite a dearth of robust evidence, may reduce adherence to evidence-based supplementation guidelines.

In low- and middle-income countries like Brazil, these concerns are amplified by social vulnerability, with lower education levels and social minority groups particularly at risk of health misinformation [18]. Socioeconomic inequalities and limited access to reproductive health information further complicate adherence and timely initiation of supplementation [19,20]. Furthermore, Brazil faces specific concerns regarding dosage, as the country’s Unified Health System (SUS—Sistema Único de Saúde) currently provides folic acid in 5000 µg tablets, more than ten times the internationally recommended daily dose of 400 µg for NTD prevention [11]. Brazil’s unique genetic diversity also raises important questions about the applicability of current international guidelines. These recommendations were derived from early clinical trials conducted among European and North American populations [21,22,23], which may not accurately reflect the metabolic and clinical responses of the Brazilian population. Together, these factors underscore the need for context-specific public health policies, more personalized dosing strategies, and evidence-based interventions adapted to Brazilian settings.

In light of these challenges, this paper aims to discuss current scientific and policy controversies surrounding folic acid supplementation, focusing on the Brazilian context. We begin with a brief historical overview and current international recommendations on folic acid use, followed by a discussion of emerging controversies and concerns related to folic acid exposure. Next, we examine Brazil’s unique challenges, including issues related to dosage, access, adherence, and genetic diversity. Finally, we propose future directions and outline short- and long-term strategies to improve the safety, effectiveness, and equity of folic acid supplementation policies. While grounded in the Brazilian context, the insights presented here may also be relevant to other low- and middle-income countries facing similar structural and population-specific barriers.

## 2. History and Current Recommendations for Folic Acid Use

Folate has been associated with reproduction as early as 1965, due to its involvement in cellular proliferation and tissue growth [24]. In 1976, Smithells et al. hypothesized that undernutrition could be a common environmental factor contributing to the occurrence of NTDs [25], prompting a series of studies on vitamin supplementation during pregnancy [26,27,28]. Subsequent research demonstrated that among the vitamins included in the multivitamin supplements, folic acid was the key component responsible for reducing the risk of NTDs [22,23]. Neural tube closure relies on complex biological processes, including convergent extension of the neural plate, neural crest cell migration, and neuroepithelial apoptosis, proliferation, and differentiation. Folate deficiency has been linked to DNA hypomethylation, impaired nucleotide synthesis, and genomic instability, mechanisms that may contribute to the development of NTDs [29].

The first randomized clinical trial (RCT) demonstrating that folic acid supplementation around the time of conception reduces the risk of NTDs was conducted in South Wales [23]. In 1991, a larger RCT including seven countries (England, Hungary, Israel, Australia, Canada, Russia, and France) reported a 72% reduction in NTD recurrence with daily supplementation of 4000 micrograms (µg) of folic acid [22]. These results led the Centers for Disease Control and Prevention (CDC) to recommend folic acid supplementation for all women of childbearing age with a previous history of NTDs [30]. The following year, a Hungarian RCT demonstrated that periconceptional supplementation with a multivitamin containing 800 µg of folic acid reduced the first occurrence of NTDs [21]. Together, these findings prompted the expansion of public health recommendations to include folic acid supplementation for all women of reproductive age [11,31,32].

Current recommendations for folic acid supplementation to prevent NTDs are still based on the initial studies conducted in the 1980s and 1990s. Both the CDC and the World Health Organization (WHO) recommend a daily intake of 400 µg of folic acid, starting before conception and continuing through the first 12 weeks of gestation [31,33]. Women at high risk for NTDs, such as those with a history of an NTD-affected pregnancy or using antifolate medications, are advised a higher dose of 4000 µg per day [31]. Since the optimal dose for preventing NTDs has never been thoroughly investigated, these agencies continue to base their guidelines on the doses used in the earliest studies: 360 µg [26,27,28], 800 µg [21], and 4000 µg for women with previous NTD-affected pregnancy [22,23]. Consequently, guidelines vary in both recommended doses and supplementation duration, as reviewed by Cawley et al. for 20 European countries [34] and as summarized in Table 1. This range of doses reflects the fact that the early studies used the formulations available at the time, which differed in folic acid content, and that higher doses were specifically tested for women at high risk of recurrence.

In the years following the initial recommendations, the expected decline in NTD prevalence was not observed [35]. This was largely attributed to the high rate of unplanned pregnancies [36], which caused many women to miss the critical window for supplementation, as neural tube closure occurs very early in embryonic development. Additionally, factors such as low levels of formal education, immigrant status, young maternal age, and absence of a partner were identified as predictors of reduced periconceptional folic acid use [37], highlighting how interventions that rely on individual lifestyle modifications are subject to socioeconomic disparities. In response to these challenges, and from a broader public health perspective, many countries have implemented mandatory folic acid food fortification programs as a population-wide preventive strategy [10].

As of July 2023, 69 countries globally have implemented mandatory folic acid fortification [10], including Brazil. The impact of these programs on NTD prevalence varies across countries. A meta-analysis across five countries (Canada, USA, Chile, Argentina, and South Africa) estimated a 46% reduction in NTD risk following the implementation of mandatory folic acid fortification [38]. Beyond NTD prevention, folic acid fortification has also been associated with reduced risks of congenital heart defects, nonsyndromic cleft lip and palate, anemia, cardiovascular disease, and certain types of cancer [39].

Nevertheless, fortification programs typically add only modest amounts of folic acid to staple foods (100–260 μg per 100 g of product), resulting in an estimated daily intake of 200 µg [39], which is below the 400 µg/day recommended by the CDC for periconceptional supplementation [31]. Therefore, even in countries with mandatory fortification policies, daily supplementation with 400 µg of folic acid is still advised for women of reproductive age. It must be emphasized that folate adequacy for preventing NTDs is critical in the first four weeks of gestation, as neural tube closure is around 28 days post-conception. However, as demonstrated in Table 2, folic acid supplementation often does not occur within the optimal window for preventing NTDs. By the time we finished the literature review, there were no other studies evaluating folic acid supplementation in Brazil.

Despite the benefits of folic acid supplementation being documented, concerns have emerged regarding the potential adverse effects of high-dose intake, including cumulative exposure from both fortified foods and supplements [13]. This issue is particularly relevant in countries like Brazil, where the public health system provides folic acid supplements at a dose of 5000 µg, ten times higher than the standard recommended intake.

## 3. Current Debates and Emerging Concerns Regarding Folic Acid Supplementation

Folate deficiency is associated with a broad spectrum of adverse health outcomes beyond NTDs, including megaloblastic anemia, chronic diseases such as cardiovascular diseases, neuropsychiatric disorders, and cancer [47]. Conversely, excessive folate intake may also be linked to adverse effects [13]. Given folate’s essential role in key biological processes, it is unsurprising that both deficiency and excess can result in a wide range of clinical manifestations. Poor folate status can lead to abnormalities in DNA and protein synthesis, post-translational modifications, and gene expression [47]. Conversely, excessive folate intake may also affect gene expression, DNA methylation patterns, uncontrolled cell growth and proliferation, modulation of inflammation and immune responses, and disruption of one-carbon metabolism [13].

Unlike 5-MTHF (and natural folate), folic acid has an established upper tolerable limit (UL), set by the US Institute of Medicine at 1000 μg/day. However, this limit was determined to prevent masking of vitamin B12 deficiency and was not based on folic acid bioavailability or metabolism [32]. Both folate and vitamin B12 deficiency can lead to megaloblastic anemia, a group of anemias characterized by abnormally large nucleated erythrocytes, caused by inhibited maturation of erythropoietic precursor cells [47]. In the 1940s, before the recognition that vitamin B12 deficiency causes pernicious anemia—a specific type of megaloblastic anemia—high-dose folic acid used for its treatment restored normal blood values but did not prevent the vitamin B12 deficiency-related neuropathy, delaying its diagnosis and leading to progressive neurological damage [47,48]. However, current medical practice does not rely on the presence of anemia for diagnosing vitamin B12 deficiency; instead, blood levels of vitamin B12 and related metabolites are directly measured [47]. Although some evidence suggests that folic acid may also exacerbate the neuropathological progression of vitamin B12 deficiency, the evidence is primarily correlative or from uncontrolled clinical observations [13]. Nonetheless, increased vigilance for identifying vitamin B12 deficiency is important in at-risk patients, such as those with low vitamin B12 intake or conditions that are associated with vitamin B12 malabsorption, and who also ingest excessive folic acid [48].

When folic acid intake exceeds the body’s metabolic capacity, unmetabolized folic acid (UMFA) can accumulate in the bloodstream due to saturation of the enzymatic conversion to its biologically active form, 5-MTHF [13]. Although no direct causal relationship has been established between elevated UMFA and adverse health outcomes, evidence suggests that UMFA may interfere with one-carbon metabolism by modulating the activity of enzymes involved in the folate cycle, potentially affecting DNA synthesis, repair, and methylation processes [13].

The impact of excessive folic acid intake on human health remains inconclusive and sometimes contradictory [13], as shown in Table 3. This suggests that both insufficient and excessive exposure to folic acid may carry potential health risks. A growing body of evidence associates folic acid excess with adverse health outcomes impacting cancer, immunity, birth outcomes, cardiovascular disease, and overall mortality [13]. A meta-analysis demonstrated that elevated serum and red blood cell folate concentrations are associated with a higher risk of developing gestational diabetes mellitus, with the effects varying depending on the timing and duration of supplementation [49]. This emphasizes the importance of considering both dose and exposure window when assessing folic acid safety. Findings from an Indian birth cohort, where pregnant women are often prescribed folic acid doses as high as 5000 µg/day, showed that high maternal folate concentrations were associated with insulin resistance in children, suggesting disturbances in fetal metabolic programming, which could contribute to a higher risk of developing diabetes later in life [50]. Additionally, misinformation stemming from the misinterpretation of scientific data has led to claims linking high folate levels to autism spectrum disorder. However, there is no evidence of a causal relationship, and meta-analyses have demonstrated a potential protective effect of periconceptional folic acid supplementation in reducing the risk of autism spectrum disorder (ASD) [51,52]. Early pregnancy is a critical period for central nervous system development. Folate deficiency during this time may impair the proliferation and migration of neural progenitor cells by altering DNA methylation and gene expression, potentially increasing the risk of ASD [53].

Irrespective of the form, folate is converted to 5-MTHF, the predominant circulating form of folate in the blood [61]. This conversion can be less efficient in individuals carrying common genetic variants in the methylenetetrahydrofolate reductase (MTHFR) gene [17]. As a result, 5-MTHF has been proposed as a potential alternative to folic acid for individuals with *MTHFR* variants [62] and is now included in several commercial multivitamin products. However, there is currently no evidence supporting the effectiveness of 5-MTHF in preventing NTDs [63]. Moreover, the CDC does not recommend *MTHFR* genotyping for clinical decision-making and states that there is no demonstrated benefit of using 5-MTHF over folic acid [64].

Therefore, international public health guidelines recommend folic acid, not 5-MTHF supplementation for NTD prevention [64,65,66]. Only a well-designed RCT directly comparing 5-MTHF and folic acid could definitively establish the efficacy of 5-MTHF for this purpose. In the meantime, the dissemination of misinformation about the superiority of 5-MTHF has led many individuals to spend substantial amounts on multivitamins containing this form, which is more expensive to manufacture than folic acid due to its lower chemical stability [67].

The optimal duration of folic acid supplementation beyond the first trimester, post-natally, and during breastfeeding remains unclear regarding its benefits to maternal and fetal health [15]. Recent studies have reported conflicting results regarding its prolonged usage. The Society of Obstetricians and Gynecologists of Canada (SOGC) recommends folic acid supplementation starting 2–3 months before conception and continuing until the end of lactation (or 4–6 weeks postpartum if not breastfeeding) [68]. Supporting this extended supplementation, a cohort study from Ireland found that continued intake of 400 µg/day of folic acid during the second and third trimesters was associated with improved neurodevelopmental outcomes in children [69]. Conversely, other studies have raised concerns about possible associations between late-pregnancy folic acid exposure and an increased risk of allergic disorders in children [70]. A prospective study from China demonstrated that initiating folic acid supplementation 1.5 months before conception, with a duration of four months, is the preferred option for preventing congenital malformation [71]. Altogether, while evidence supports both extended and more limited durations of folic acid use, further studies are needed to clarify the long-term benefits and potential risks associated with supplementation beyond the first trimester.

**Figure 1:**
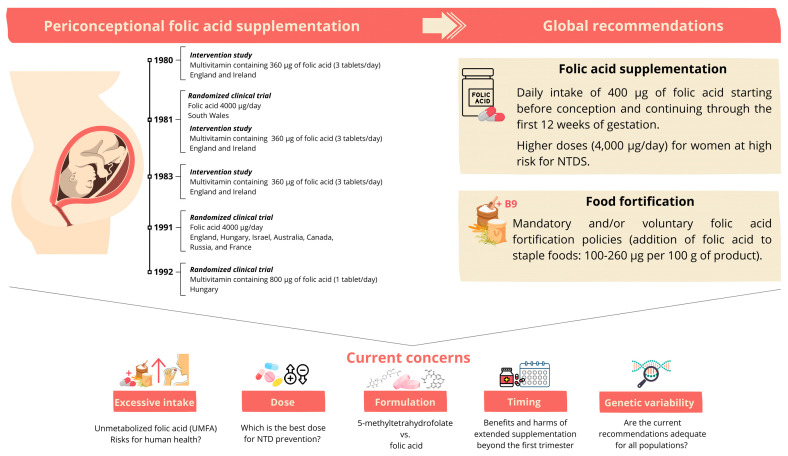
Timeline of folic acid supplementation, current public health recommendations, and emerging concerns. The left panel summarizes key randomized control trials (RCTs) and intervention studies from 1980 to 1992 that established the protective effect of folic acid against neural tube defects (NTDs) [21,22,23,26,27,28]. The right side summarizes current global recommendations, including daily supplementation and food fortification policies [31,33,39]. At the bottom, ongoing concerns are illustrated, such as excessive intake, dose optimization, formulation differences, the ideal timing of supplementation, and genetic variability.

Hence, while folate adequacy is essential in early gestation, the optimal duration, dosage, and long-term implications of supplementation warrant further investigation. Future research, including RCT comparing folic acid and 5-MTHF, different dosing regimens, and durations of use, is essential to guide evidence-based recommendations that maximize efficacy in NTD prevention while protecting overall maternal–child health. Figure 1 summarizes the evolution of folic acid supplementation, from early clinical trials to current guidelines and emerging challenges.

Although most of the available evidence and recommendations stem from high-income countries, the local context must also be considered. In Brazil, specific challenges related to the available formulations, health system policies, and socioeconomic disparities further complicate adherence to folic acid supplementation.

## 4. Specific Aspects of Folic Acid Supplementation in Lower-Income Countries: The Brazilian Example

Brazil is a vast and diverse country characterized by pronounced socioeconomic disparities and significant inequalities in health and nutrition [72]. Although the public health system, SUS, provides universal healthcare coverage and serves approximately 60% of the population, access to health services and health outcomes vary considerably across regions [73,74]. Populations with lower incomes and those residing in economically deprived areas are the most disadvantaged [75]. These inequalities also influence dietary patterns: consumption of fresh fruits and vegetables declines progressively from higher- to lower-income strata and is lower among Black individuals compared to White individuals [76], potentially limiting natural folate intake in these groups. Furthermore, adherence to folic acid supplementation is associated with maternal education and socioeconomic status [20,77,78], underscoring how folate availability from both dietary and supplemental sources is shaped by regional, racial, social, and economic factors in Brazil.

Acquired folate deficiency is associated with reduced intake, chronic alcohol consumption, increased demands in pregnancy, and malabsorption [2]. These risk factors are often exacerbated in socioeconomically disadvantaged populations, where individuals may face limited access to a variety of nutrient-rich foods. In such environments, the lack of clean water, sanitation, and hygiene further increases the risk of diarrheal diseases [79], which can compromise the absorption of essential nutrients, including folate. Moreover, lower socioeconomic status is associated with greater alcohol-attributable health harm [80]. Therefore, these vulnerable populations are more likely to have inadequate folate levels, emphasizing the need for targeted public health policies to mitigate deficiencies and their broader health consequences.

Beyond these contextual disparities, current recommendations for folic acid supplementation are based on the early clinical trials conducted in European and North American populations [21,22,23]. Such guidelines may not fully reflect the reality of genetically admixed populations such as Brazil. A recent study has identified Brazilians as among the most genetically diverse populations worldwide, with a complex ancestry comprising European, African, and Indigenous contributions [81]. This diversity includes genetic variants that may influence nutrient metabolism and drug response [81]. Genetic polymorphisms or mutations in proteins involved in one-carbon metabolism (e.g., MTHFR) or folate transport (e.g., the reduced folate carrier) can modulate folate metabolism and increase susceptibility to folate-related diseases [82,83]. The frequency and distribution of these variants vary across populations and ethnic groups [84].

In admixed populations like Brazil, this genetic variability may lead to differences in individual responses to folic acid supplementation. For example, individuals carrying certain *MTHFR* polymorphisms may have reduced enzymatic activity, resulting in lower conversion of folate to its active forms [82]. Similarly, variants in folate transport proteins may influence cellular uptake, bioavailability, and tissue distribution of folate, affecting the risk of deficiency [85]. The unique genetic admixture of the Brazilian population, shaped by complex history and migration patterns, has revealed a significant proportion of previously unreported genetic variants [84], underscoring the importance of considering Brazil’s unique genetic profile when developing public health strategies, including those involving folic acid supplementation.

In response to the burden of NTDs, Brazil implemented a nationwide fortification policy in 2002, mandating the addition of folic acid to wheat and corn flours [86]. According to the current regulation established by the National Health Surveillance Agency, the required folic acid content ranges from 140 to 220 µg per 100 g of product [87]. However, data from national flour fortification programs have shown that more than half of the products fail to meet the recommended levels. In 2021, 50% of the products analyzed exceeded the upper limit, while 12% fell below the minimum threshold [88]. Notwithstanding these challenges, the policy has been associated with a 30% reduction in NTD prevalence and a 22.8% decrease in related healthcare costs [89]. Notably, one study reported a comparatively lower effect of folic acid fortification on NTD prevention in Brazil relative to other Latin American countries, attributing this to factors such as Brazil’s unique ethnic composition, differences in dietary intake, and variability in the folic acid levels of fortified products [90]. This underscores the importance of daily periconceptional supplementation with 400 µg of folic acid, as fortification alone may be insufficient to achieve optimal preventive levels.

The public health system provides folic acid in two formulations: a 200 µg/mL liquid solution and 5000 µg tablets, both included in the Brazilian National Essential Medicines List [91]. To achieve the recommended daily intake of 400 µg during the periconceptional period, women must take 40 drops of the liquid formulation, an inconvenient regimen that raises concerns about adherence. Unpublished data from our research group indicate that the 5000 µg tablet originally intended for high-risk groups has been frequently prescribed, regardless of individual risk profiles (Ethical Approval No. 79644924.0.0000.5327).

This widespread use of high-dose folic acid supplementation raises concerns about overexposure and potential adverse health effects. A non-controlled intervention study with 30 healthy Brazilian adults showed that daily folic acid supplementation at a 5000 µg dose for 90 days resulted in elevated circulating concentrations of UMFA [92]. Similar findings were reported in a case–control study involving patients with hereditary spherocytosis [93].

While some women may be exposed to high doses of folic acid, a substantial proportion receive no supplementation during the most critical period [20]. Most pregnancies in Brazil are unplanned [94], and many women fail to initiate supplementation within the periconceptional window [20]. This results in two contrasting scenarios: inadequate folic acid intake during key stages of fetal development in some women, and excessive intake without clinical justification in others. A study conducted at a hospital in southern Brazil reported that only 1.6% of women followed national recommendations regarding dosage and timing [20]. Another study found that only 8.9% of Brazilian women used folic acid-containing supplements during the first trimester of pregnancy [95].

Another challenge stemming from the inconsistency between national recommendations and the folic acid formulations available in the public health system is the adoption of alternative regimens by clinicians. As observed in the unpublished data from our group, some healthcare providers prescribe the 5000 µg tablet at non-daily intervals. However, given that folic acid is a water-soluble vitamin and is not stored in large amounts [96], such strategies may lead to suboptimal bioavailability. This gap has also encouraged recommendations for commercial multivitamins not supplied by the public health system. These products are often expensive for many women, and, in some cases, the costs of purchasing these supplements may divert limited financial resources away from other essential needs, such as nutritious food. Additionally, most commercial supplements now contain 5-MTHF instead of folic acid, despite the lack of evidence supporting 5-MTHF’s effectiveness in preventing NTDs [63].

These complex and sometimes contradictory realities underscore the urgent need for more precise, accessible, and equitable public health policies. Improved training and education for healthcare providers is also essential to ensure that folic acid recommendations are both effective and appropriately tailored to Brazil’s unique social, economic, and genetic landscape.

## 5. Future Directions

### 5.1. Directions for Brazil

Both short- and long-term strategies are needed to ensure folic acid supplementation is effective, safe, and equitable in Brazil. In the short term, efforts should focus on strengthening the training and awareness of healthcare providers, particularly those in primary care and those prescribing medications known to interfere with folate metabolism (e.g., anticonvulsants, methotrexate, sulfasalazine) [97]. Developing a specific national guideline focused on folic acid supplementation would be a crucial step toward standardizing clinical practice. In parallel, public health campaigns tailored to Brazil’s diverse sociocultural and economic contexts are necessary to promote the importance of consistent supplementation, before and through the first trimester of pregnancy. These campaigns should address accessibility barriers, low literacy levels, and regional disparities. In areas with limited access to healthcare, initiatives that bring healthcare providers directly to schools, community centers, and homes should be prioritized. Training health professionals to communicate the benefits of folic acid supplementation in a clear and approachable manner is also essential, using visual aids, videos, and face-to-face interactions to enhance understanding and engagement.

In the long term, the public health system should incorporate user-friendly formulations, such as 400 µg folic acid tablets, which align with national recommendations and could improve adherence by simplifying the supplementation regimen. To support this strategy, studies assessing the cost-effectiveness and budgetary impact of including the 400 µg formulation in the SUS are needed. Broader access to the appropriate dose formulation could also reduce the growing use of alternative regimens. These studies should comprehensively evaluate the impact of increased folic acid supplementation adherence on the prevention of NTDs and other folate-related conditions, as well as the associated healthcare cost savings. These savings include avoided expenses for surgical interventions, hospital admissions, long-term rehabilitation, and productivity losses from family members. Direct program costs, such as medication procurement, distribution, and storage, must also be considered. Additionally, cost-effectiveness analyses should model different scenarios of adherence rates and regional disparities. Post-implementation, robust monitoring and evaluation systems will be necessary to assess whether the inclusion of the 400 µg formulation in the SUS translates into measurable improvements in maternal and child health outcomes and generates economic benefits for the healthcare system.

In parallel with improved supplementation strategies, effective monitoring systems are needed to assess folate status at the population level. This is especially critical in countries with folic acid fortification policies, such as Brazil, where actual intake varies due to individual dietary patterns. Because folic acid intake from fortified foods cannot be precisely estimated, biomarker-based surveillance is essential for accurately evaluating folate levels. Additionally, stricter regulatory oversight is necessary to ensure that fortified flours consistently meet the legally mandated folic acid levels, as many products currently fail to meet these requirements [88].

Therefore, investment in national surveillance systems to monitor blood folate concentrations, supplementation coverage, and NTD outcomes is critical to support more effective and targeted public health interventions. Locally driven research initiatives are also critical. These should focus on generating evidence for optimal supplementation strategies in the Brazilian context, including dose–response assessments, and evaluating common genetic polymorphisms in folate metabolism enzymes, such as the *MTHFR* gene. These efforts would support the development of a national guideline that considers Brazil’s unique demographic, clinical, and genetic characteristics.

### 5.2. Global Directions

Many of the challenges faced by Brazil are similar in other low- and middle-income countries, where socioeconomic vulnerabilities, limited access to healthcare, and lower education levels can impair adherence to folic acid supplementation [77,98]. Globally, additional challenges include public confusion around supplementation regimens, particularly regarding the use of 5-MTHF and the impact of misinformation. Sensationalized media coverage and misinterpretation of scientific findings have fueled unfounded claims, such as the notion that high folate levels cause autism spectrum disorder or that 5-MTHF is universally superior to folic acid, despite the lack of supporting evidence. These narratives erode public trust and reduce adherence to evidence-based recommendations. Addressing these issues requires coordinated efforts in science communication, digital literacy, and developing accessible, transparent public health messaging.

**Figure 2:**
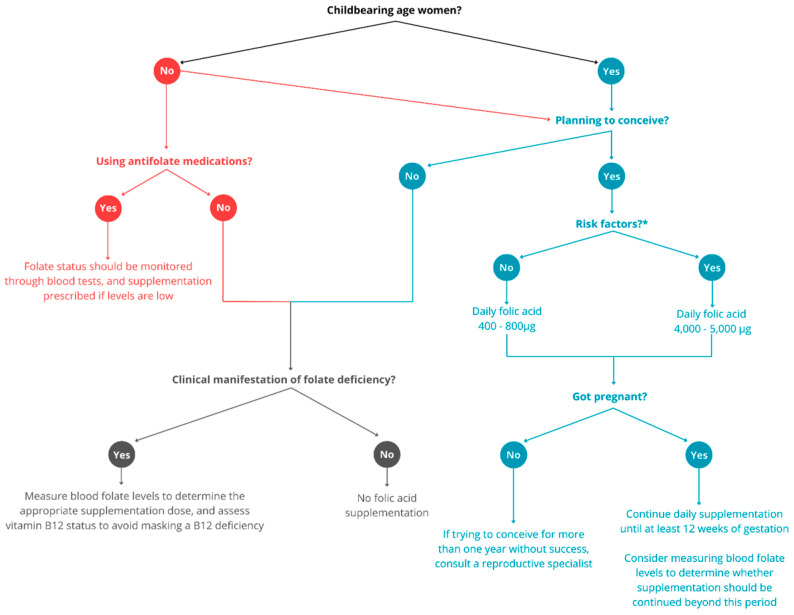
Clinical decision tree for folic acid supplementation focused on the prevention of neural tube defects. * risk factors include previous NTD-affected pregnancy and the use of antifolate drugs (e.g., anticonvulsants, methotrexate, and sulfonamides). Patients who are not planning to conceive should be using contraception. Women not of childbearing age are represented in red, women of childbearing age in blue, and women who are neither planning to conceive nor using antifolate medications in grey.

Another global concern relates to the high prevalence of unplanned pregnancies [36] and the increasing use of folate-depleting medications, such as anticonvulsants prescribed not only for epilepsy but also for mood disorders and migraine [99]. In light of this, routine folic acid supplementation should be considered for all women of reproductive age who are prescribed such medications. This emphasizes the need for clear, updated clinical guidelines and improved training for healthcare providers on optimal dosage, timing, and target populations. Figure 2 presents a clinical decision tree for folic acid supplementation.

Ultimately, personalized approaches to folic acid supplementation may be considered. Countries like Canada, for example, have adopted targeted screening of serum or red blood cell folate levels in specific groups, such as women planning pregnancy, those already pregnant, or those taking medications with antifolate effects [100]. This personalized approach may inform tailored supplementation strategies, including adjustments of dose throughout pregnancy, thus supporting more precise clinical decision-making.

**Figure 3:**
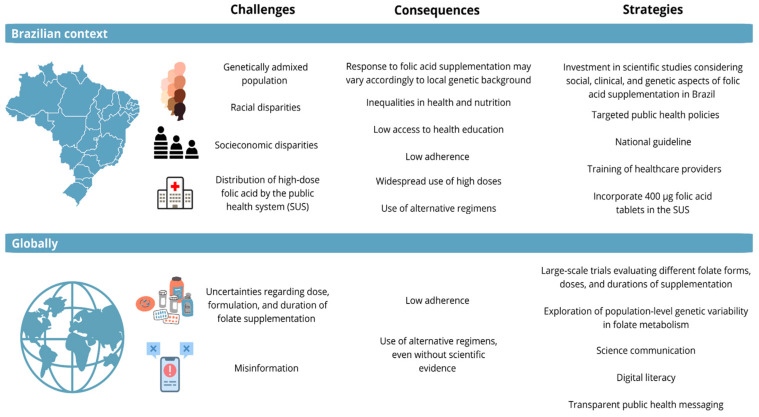
Brazilian and global challenges in folic acid supplementation. This figure summarizes the main challenges related to folic acid supplementation and possible strategies to mitigate their consequences.

A global research agenda is needed to fill critical knowledge gaps, particularly in underrepresented populations. This includes large-scale trials evaluating different folate forms, studies on the impact of extended supplementation during pregnancy, and exploration of population-level genetic variability in folate metabolism. Together, these national and international strategies can help bridge the gap between evidence and policy, ensuring that folic acid supplementation is implemented in a way that is both effective and equitable across diverse global contexts. Figure 3 highlights the main challenges, consequences, and future directions of folic acid supplementation in both the Brazilian and global contexts.

## 6. Conclusions

Both insufficient and excessive folic acid exposures raise important concerns for maternal–child health globally. In parallel, alternative folate formulations, such as 5-MTHF, have been commercialized despite a lack of clinical evidence supporting their efficacy in preventing NTDs, underscoring the urgent need for robust clinical trials to evaluate the effectiveness of different regimens. While this discussion centers on Brazil, many of the challenges and proposed strategies are equally relevant to other low- and middle-income countries facing similar structural, socioeconomic, and genetic diversity barriers. These insights may therefore inform broader international efforts to optimize supplementation policies and practices. Ultimately, there is a pressing need to reassess current strategies through the lens of population-specific data, genetic variability, and optimal dosing and timing. This approach should not only aim to enhance the effectiveness and equity of NTD prevention but also to realize the broader health benefits of adequate folate status throughout the reproductive period and early life.

## Figures and Tables

**Table 1:** Folic acid supplementation guidelines to prevent neural tube defects.

Organization	Place	Year	Recommendations
U.S. Preventive Services Task Force	United States	2023	Population: all persons planning to or who could become pregnant General dose: daily 400 to 800 μg High-risk dose: NA Duration: start at least 1 month prior to conception and continue through the first 2 to 3 months of pregnancy
Society of Obstetricians and Gynaecologists of Canada (SOGC)	Canada	2022	Population: Women aged 12–45 years who could become pregnant General dose: daily 400 μg High-risk dose: daily 4000 to 5000 μg * Duration: at least 2–3 months before conception, throughout the pregnancy, and for 4–6 weeks postpartum or as long as breastfeeding continues
Royal Australian and New Zealand College of Obstetricians and Gynaecologists (RANZCOG)	Australia, New Zealand	2024	Population: women planning to become pregnant General dose: daily 400 to 800 μg High-risk dose: daily 5000 μg Duration: at least four weeks before intended pregnancy and continues for the first 12 weeks of pregnancy
Federación Latinoamericana de Sociedades de Obstetricia y Ginecología (FLASOG)	Latin America	2020	Population: women planning to become pregnant General dose: daily 400 μg High risk dose: daily 4000 μg Duration: 3 months before conception and continues for the first 12 weeks of pregnancy

* requires two separate periods of supplementation: from pre-conception to 12 weeks of gestation, and from 12 weeks of gestation until completion of breastfeeding, when the folic acid supplementation dosage reverts to the low-dosage regimen; NA (not available).

**Table 2:** Patterns of folic acid supplementation among women based on cross-sectional studies conducted in low- and middle-income countries.

Place	Period	Participants	Folic Acid Supplementation
São Paulo, Brazil	May 2004–May 2005	Pregnant women (*N* = 250)	Periconceptional start: 77 (30.8%); start at two months of gestation: 43 (17.2%) [40]
Rio Grande do Sul, Brazil	April–August 2006	Postpartum women (*N* = 1450)	Start at some point during pregnancy: 461 (31.8%); periconceptional start: 62 (4.3%) [41]
Rio Grande do Sul, Brazil	January 2016–January 2017	Postpartum women (*N* = 765)	Start before pregnancy: 27 (3.5%), in the first trimester: 292 (38.2%), second trimester: 57 (7.4%), and third trimester: 9 (1.2%) [20]
Golestan, Iran	June–November 2008	Pregnant women (*N* = 676)	Periconceptional start: 136 (20.1%) [42]
Jos, Nigeria	January–July 2012	Pregnant women (*N* = 543)	Periconceptional start: 26 (4.8%); among women with prior NTD-affected pregnancy, 6/15 (40%) used folic acid during the periconceptional period [43]
Jimma, Ethiopia	February–March 2012	Pregnant women (*N* = 339)	Start at some point during pregnancy: 3 (0.9%) [44]
Anhui, China	October 2008–September 2009	Pregnant women (*N* = 4290)	Start before pregnancy: 1405 (33%); start in early pregnancy: 2797 (65.2%); periconceptional start: 2905 (67.7%); taken throughout the correct period: 690 (16.1%) [45]
China	June–December 2016	Pregnant women (*N* = 428)	Start before pregnancy: 198 (46.3%) [46]

Data include study location, period, participant type, sample size, and timing of folic acid supplementation initiation. Values are presented as absolute numbers and percentages based on each study’s sample. References are indicated in brackets and correspond to the numbered bibliography.

**Table 3:** Benefits and potential risks associated with excessive folate intake.

Benefits	Potential Risks
Prevention of neural tube defect recurrence [23] Congenital heart defects prevention [54] Protection against preterm birth and low birth weight [55] Enhanced communication skills in children [55]	Increased cancer risk in the presence of preneoplastic lesions [56] Mask B12 deficiency anemia [57] Gestational diabetes mellitus [49] Low birth weight [58] Insulin resistance in children [50] Impaired neurocognitive development in children [59] Increased risk of childhood asthma [60]

This table summarizes the reported benefits and potential adverse outcomes associated with excessive folate intake. References are indicated in brackets and correspond to the numbered bibliography.

## Data Availability

Data availability statement is not applicable.

## Abbreviations

The following abbreviations are used in this manuscript:

CDC	Center for Disease Control and Prevention
MTHFR	Methylenetetrahydrofolate reductase
5-MTHF	5-methyltetrahydrofolate
NTD	Neural tube defect
RCT	Randomized clinical trial
SUS	Sistema Único de Saúde (Unified Health System)
UMFA	Unmetabolized folic acid
